# Is ChatGPT a Reliable Tool for Explaining Medical Terms?

**DOI:** 10.7759/cureus.77258

**Published:** 2025-01-10

**Authors:** Ayse Dilara Oztermeli

**Affiliations:** 1 Emergency Medicine, Derince State Hospital, Kocaeli, TUR

**Keywords:** artificial intelligence, chatgpt, clinical decision support, medical terminology, patient education

## Abstract

Background

The increasing reliance on the internet for health-related information has driven interest in artificial intelligence (AI) applications in healthcare. ChatGPT has demonstrated strong performance in medical exams, raising questions about its potential use in patient education. However, no prior study has evaluated the reliability of ChatGPT in explaining medical terms. This study investigates whether ChatGPT-4 is a reliable tool for translating frequently used medical terms into language that patients can understand.

Methodology

A total of 105 frequently used medical terms were selected from the University of San Diego's medical terminology list. Four groups - general practitioners, resident physicians, specialist physicians, and ChatGPT-4 - were tasked with defining these terms. Responses were classified as correct or incorrect. Statistical analyses, including chi-square and post-hoc tests, were conducted to compare accuracy rates across groups.

Results

ChatGPT-4 achieved a 100% accuracy rate, outperforming specialist physicians (98.1%), resident physicians (93.3%), and general practitioners (84.8%). The differences in accuracy rates between groups were statistically significant (χ²=25.99, p<0.00001). Post-hoc analyses confirmed significant pairwise differences, such as ChatGPT-4 vs. specialist physicians (p<0.001) and specialist physicians vs. resident physicians (p=0.02).

Conclusions

ChatGPT-4 demonstrated superior reliability in translating medical terms into understandable language, surpassing even highly experienced physicians. These findings suggest that ChatGPT could be a valuable auxiliary tool for improving patient comprehension of medical terminology. Nonetheless, the importance of consulting healthcare professionals for clinical decision-making remains crucial.

## Introduction

In recent years, the rate of patients turning to the Internet for their health concerns has significantly increased. During this process, interest in the use of artificial intelligence applications in the healthcare field has been growing. Particularly, ChatGPT's success in medical exams has sparked discussions on whether artificial intelligence could serve as an effective tool in addressing health problems [1-5].

Following ChatGPT's demonstrated success in medical exams, research has intensified on its potential utility in directly addressing health problems. In a study conducted by Eraybar et al., it was revealed that ChatGPT could be utilized to facilitate faster decision-making in emergency triage processes [6]. Additionally, research has demonstrated ChatGPT's success in diagnosing conditions such as pediatric familial Mediterranean fever, otological diseases, rhabdomyosarcoma, and thoracic radiological cases [7].

The majority of these studies have focused on ChatGPT's potential use as an "artificial intelligence doctor." On the other hand, it is also known that patients attempt to use the internet as a resource to better understand their health problems. Before the widespread adoption of artificial intelligence technologies, it was known that patients often tried to comprehend their conditions by watching videos on platforms like YouTube, and various studies have been conducted regarding the reliability of these platforms [8-11]. Similar studies are currently being conducted on ChatGPT.

Patients also turn to the Internet when they do not fully understand the medical terminology used by their doctors. This may occur when attempting to interpret documents such as radiological evaluation reports or pathology reports. However, no previous study in the literature has questioned the reliability of ChatGPT in explaining medical terms. In this study, we aim to investigate whether ChatGPT is a reliable tool for translating frequently used medical terms into language that patients can understand.

## Materials and methods

Study design and participants

This study utilized a comparative, cross-sectional design involving four groups: general practitioners, resident physicians, specialist physicians, and ChatGPT-4. Participants were recruited with the following inclusion and exclusion criteria:

The inclusion criteria for the study encompassed general practitioners with a minimum of one year of clinical experience, resident physicians actively undergoing specialization training, and specialist physicians who had completed board certification and were engaged in active clinical practice.

The exclusion criteria for the study included physicians with no clinical experience and participants who failed to complete the assessment process.

Data collection

A total of 105 frequently used medical terms were selected from the University of San Diego’s Professional and Continuing Education (PCE) program’s online resource (https://pce.sandiego.edu/medical-terminology-list/).

Participants were asked to define each medical term in a written format without external resources. The same set of terms was entered into ChatGPT-4o, an advanced AI model developed by OpenAI, under identical conditions. Each response was evaluated as either "correct" or "incorrect" by an independent panel of two senior clinicians to ensure accuracy and eliminate bias.

Outcome measures

The primary outcome measure was the accuracy rate, calculated as the number of correctly defined terms divided by the total number of terms (105).

Statistical analysis

The data collected from each group were analyzed by calculating the number of correct and incorrect responses for the 105 medical terms. Accuracy rates were then determined for each group, serving as the dependent variable. The groups - ChatGPT-4, specialist physicians, resident physicians, and general practitioners - were treated as the independent variable.

The differences in accuracy rates among groups were assessed using the Chi-square test, which is appropriate for analyzing categorical data. Specifically, this test was chosen to evaluate whether the proportions of correct responses significantly differed among the groups.

To identify pairwise differences between groups, Bonferroni-adjusted post-hoc comparisons were performed. This adjustment was chosen to control for Type I errors arising from multiple comparisons, ensuring the validity of the results.

The analysis aimed to determine whether the accuracy rates of ChatGPT-4, specialist physicians, resident physicians, and general practitioners were significantly different. All statistical analyses were conducted using Jamovi 2.3.19.0. Results were presented as proportions, and p-values were reported to indicate the level of statistical significance in group comparisons.

Ethical considerations

The study was conducted in accordance with ethical principles outlined in the Declaration of Helsinki. Written informed consent was obtained from all participating physicians. As the study involved publicly available AI models, no additional ethical approval was required for ChatGPT-4.

## Results

The 105 frequently used medical terms were evaluated across four groups. The accuracy rates for each group were as follows:

General practitioners correctly answered 89 terms (84.8%) and incorrectly answered 16 terms.

Resident physicians correctly answered 98 terms (93.3%) and incorrectly answered seven terms.

Specialist physicians correctly answered 103 terms (98.1%) and incorrectly answered two terms.

ChatGPT-4 correctly answered all 105 terms, achieving a 100% accuracy rate (Table 1).

**Table 1:** Performance comparison of four groups in defining medical terms

Group	Correct Answers	Incorrect Answers	Accuracy Rate (%)
General Practitioners	89	16	84.8
Resident Physicians	98	7	93.3
Specialist Physicians	103	2	98.1
ChatGPT-4	105	0	100.0

The findings of this study demonstrate the superiority of ChatGPT-4 over human groups in interpreting frequently used medical terms. The statistically significant difference in accuracy rates among groups (χ²=25.99, p<0.00001) indicates that the performance of ChatGPT-4 is not due to chance but reflects a systematic advantage.

Post-hoc test results revealed that all pairwise comparisons between the groups were statistically significant (Table 2).

**Table 2:** Post-hoc comparisons of accuracy rates between groups with corresponding p-values.

Group Comparison	p-value
ChatGPT-4 vs Specialist Doctors	<0.001
ChatGPT-4 vs Resident Doctors	<0.001
ChatGPT-4 vs General Practitioners	<0.001
Specialist Doctors vs Resident Doctors	0.02
Specialist Doctors vs General Practitioners	<0.001
Resident Doctors vs General Practitioners	0.03

These results strongly support the superior performance of ChatGPT-4. Additionally, the differences in performance among human groups reflect variations in clinical experience and educational level, with specialist doctors outperforming residents and residents outperforming general practitioners.

## Discussion

Our study demonstrated that ChatGPT is highly reliable in explaining frequently used medical terminology, outperforming even specialist physicians with a 100% accuracy rate. To our knowledge, this study is the first to compare ChatGPT with clinicians in terms of mastery of medical terminology in the literature.

Our findings revealed that even human doctors can make errors in understanding medical terminology. Although specialist physicians made relatively fewer mistakes, they were occasionally outperformed by ChatGPT. This may be explained by the fact that some of the terms used in our study are specific to certain specialties. These findings suggest that doctors themselves could use ChatGPT as a medical dictionary.

Among the human participants, it was found that specialist physicians performed better than resident doctors and resident doctors outperformed general practitioners. This result indicates that knowledge levels increase with seniority and experience. It highlights that clinical experience and specialization training enhance knowledge of medical terminology. However, the fact that this training still falls short compared to an AI model underscores the potential importance of AI-supported educational tools.

The idea of using ChatGPT in the medical field initially emerged from its success in medical exams [4, 5]. Over time, research has focused on more specific topics, and its success rates in diagnosing certain diseases have been questioned. For instance, a study by La Bella et al. demonstrated ChatGPT's reliability in addressing pediatric familial Mediterranean fever cases [12]. This study concluded that ChatGPT performed well in this specific context. Similarly, in a study by Cesur and Güneş, ChatGPT was asked about radiological images of thoracic diseases, achieving an accuracy rate of 59.7% [13]. However, these studies also revealed that ChatGPT’s responses never fully reached the level of expertise. Therefore, it is clear that patients relying solely on AI for decision-making could be misled. This study focuses on medical terminology to examine whether using ChatGPT to understand terms mentioned in expert opinions could be misleading. Our results indicate that ChatGPT is a highly reliable method for understanding medical terminology.

It is well-known that patients turn to the Internet for information about their health problems. Previous studies have shown that non-AI-powered websites are not reliable resources. Studies involving ChatGPT have mostly focused on how doctors can benefit from this AI [14]. However, there is no study in the literature evaluating whether patients can reliably use ChatGPT to gain information. Our study shows that patients can easily use ChatGPT to understand information such as expert opinions, pathology, and radiology reports. While simplifying medical terms provides valuable information to patients, it remains essential to consult experts for final decisions regarding health problems.

Our study has some limitations. Firstly, thousands of medical terms are used among doctors, but only a subset was selected for this study. Additionally, although most of the terms have Latin origins, variations may occur in different countries. Only English terms were used in this study. Lastly, the study evaluated individual terms rather than entire reports or expert opinions. However, understanding individual terms is thought to contribute to comprehending entire reports.

## Conclusions

In conclusion, this study demonstrates that ChatGPT-4 is a highly accurate and reliable tool for explaining medical terminology, surpassing the performance of general practitioners, resident physicians, and even specialist physicians. While AI tools like ChatGPT hold promise for improving patient comprehension, they should be viewed as supportive resources rather than replacements for expert clinical guidance. Integrating AI into healthcare can help bridge gaps in communication, empowering patients to better understand their health while maintaining the irreplaceable role of healthcare professionals.
